# Accuracy of Event Rate and Effect Size Estimation in Major Cardiovascular Trials: A Systematic Review

**DOI:** 10.1001/jamanetworkopen.2024.8818

**Published:** 2024-04-30

**Authors:** Christoph B. Olivier, Lasse Struß, Nathalie Sünnen, Klaus Kaier, Lukas A. Heger, Dirk Westermann, Joerg J. Meerpohl, Kenneth W. Mahaffey

**Affiliations:** 1 Department of Cardiology and Angiology, Cardiovascular Clinical Research Center, University Heart Center Freiburg–Bad Krozingen, Faculty of Medicine, University of Freiburg, Freiburg, Germany; 2 Institute of Medical Biometry and Medical Informatics, University Medical Center Freiburg, Faculty of Medicine, University of Freiburg, Freiburg, Germany; 3 Institute for Evidence in Medicine, University Medical Center Freiburg, Faculty of Medicine, University of Freiburg, Freiburg, Germany; 4 Cochrane Germany, Cochrane Germany Foundation, University of Freiburg, Freiburg, Germany; 5 Department of Medicine, Stanford Center for Clinical Research, Stanford University School of Medicine, Stanford, California

## Abstract

**Importance:**

For the design of a randomized clinical trial (RCT), estimation of the expected event rate and effect size of an intervention is needed to calculate the sample size. Overestimation may lead to an underpowered trial.

**Objective:**

To evaluate the accuracy of published estimates of event rate and effect size in contemporary cardiovascular RCTs.

**Evidence Review:**

A systematic search was conducted in MEDLINE for multicenter cardiovascular RCTs associated with MeSH (Medical Subject Headings) terms for cardiovascular diseases published in the *New England Journal of Medicine*, *JAMA*, or the *Lancet* between January 1, 2010, and December 31, 2019. Identified trials underwent abstract review; eligible trials then underwent full review, and those with insufficiently reported data were excluded. Data were extracted from the original publication or the study protocol, and a random-effects model was used for data pooling. This review was conducted according to the Preferred Reporting Items for Systematic Reviews and Meta-Analyses reporting guideline. The primary outcome was the accuracy of event rate and effect size estimation. Accuracy was determined by comparing the observed event rate in the control group and the effect size with their hypothesized values. Linear regression was used to determine the association between estimation accuracy and trial characteristics.

**Findings:**

Of the 873 RCTs identified, 374 underwent full review and 30 were subsequently excluded, resulting in 344 trials for analysis. The median observed event rate was 9.0% (IQR, 4.3% to 21.4%), which was significantly lower than the estimated event rate of 11.0% (IQR, 6.0% to 25.0%) with a median deviation of −12.3% (95% CI, −16.4% to −5.6%; *P* < .001). More than half of the trials (196 [61.1%]) overestimated the expected event rate. Accuracy of event rate estimation was associated with a higher likelihood of refuting the null hypothesis (0.13 [95% CI, 0.01 to 0.25]; *P* = .03). The median observed effect size in superiority trials was 0.91 (IQR, 0.74 to 0.99), which was significantly lower than the estimated effect size of 0.72 (IQR, 0.60 to 0.80), indicating a median overestimation of 23.1% (95% CI, 17.9% to 28.3%). A total of 216 trials (82.1%) overestimated the effect size.

**Conclusions and Relevance:**

In this systematic review of contemporary cardiovascular RCTs, event rates of the primary end point and effect sizes of an intervention were frequently overestimated. This overestimation may have contributed to the inability to adequately test the trial hypothesis.

## Key Points

**Question:**

How accurate are estimated event rates and effect sizes in contemporary cardiovascular randomized clinical trials (RCTs)?

**Findings:**

In this systematic review of 344 contemporary cardiovascular RCTs, overestimation of event rates was common. In addition, 4 of 5 trials overestimated the effect size of the tested intervention.

**Meaning:**

These findings suggest that event rates and effect sizes in contemporary cardiovascular RCTs are frequently overestimated, which may contribute to the inability to answer the trial hypothesis.

## Teaser

This systematic review examines the accuracy of published estimates of event rates and effect sizes in contemporary cardiovascular randomized clinical trials.

## Introduction

Only 1 in 10 pharmaceutical development programs that enter clinical testing support approval of new drugs by the US Food and Drug Administration.^1^ This high failure rate is considered one of the major contributors to the capitalized research and development costs of approximately $1 billion to bring a new drug to market.^2^ The majority of trials fail to reject the null hypothesis.^3^ Reports often include inconclusive results due to potential type II errors. Underpowered studies may prevent advances in patient care, and they also require valuable resources to design and conduct and may compete with other trials for the same population.

During the design of a randomized clinical trial (RCT), estimation of the expected event rate and effect size is a key component to calculating the sample size. Overly optimistic estimation of event rates and effect sizes may lead to underpowered trials. This study aimed to (1) evaluate event rate (control group) and effect size estimation accuracy in contemporary cardiovascular RCTs and (2) identify factors associated with higher accuracy of event rate and effect size estimation.

## Methods

This systematic review followed the Preferred Reporting Items for Systematic Reviews and Meta-Analyses (PRISMA) reporting guideline.

### Literature Search

The MEDLINE database was queried for RCTs published between January 1, 2010, and December 31, 2019, in the *New England Journal of Medicine*, *JAMA*, or the *Lancet*, associated with MeSH (Medical Subject Headings) terms for cardiovascular diseases (unique MeSH identifier: D002318). We selected these top 3 journals in the general and internal medicine category, per the Clarivate Journal Citation Reports^4^ based on the highest impact factor. The results of the literature search are presented in eTable 1 in Supplement 1.

### Screening and Variable Extraction

Abstracts were screened for inclusion and exclusion criteria. Only multicenter RCTs with a dichotomous primary end point, with only 2 study groups, and with more than 100 participants were included. Data were extracted from the original publication, associated study protocol, or publication of the study design and rationale. Additional information, such as sponsorship or funding, was acquired through international clinical trial registries such as ClinicalTrials.gov. Inclusion and exclusion criteria are listed in eTable 2 in Supplement 1. The identified articles and protocols underwent full review for data extraction. If the trial did not provide the estimated event rate and effect size or was identified as proof of concept, it was excluded from analysis.

### Event Rate and Effect Size Measure

To determine estimation accuracy, we compared the observed to the hypothesized event rate (control group) and the effect size, respectively. Estimated and observed event rates were extracted for the same period, with time fixed for the estimated event rate as the reference. If the time duration differed between estimated and observed event rates, harmonization in the trial was achieved by extracting the observed event rate from the Kaplan-Meier curve corresponding to the time for the estimated event rate with DigitizeIt specialized software, version 2.5 (DigitizeIt).^5^ For trials that defined the primary end point as event-free survival, we converted the event rate by calculating 1 minus the indicated rate for event-free survival. To harmonize trials that tested the superiority of an intervention, effect size measures (ie, hazard ratio, odds ratio, or risk ratio) were summarized as the effect size. If the effect size measures of the estimation and observation did not correspond, the estimated effect size measures were converted to the measurement of the observation (eg, relative risk to hazard ratio; formulas are provided in the eAppendix in Supplement 1).^6^

### Variable Definitions and Selection

#### General Characteristics

A research organization was defined as the group that conducted the trial operations and included academic groups or contract research organizations. We extracted whether the trial was industry sponsored or investigator initiated. For investigator-initiated trials, we determined the funding source as co-funded by industry or solely funded by nonprofit organizations (eg, foundations) or by public funding. All interventions that were not a drug or device were classified as other trials (eg, treatment strategies).

#### Design Aspects

If the participant, investigator, or both were blinded to the assigned intervention, the trial was labeled as masked. Trials were categorized according to whether a clinical events committee was installed for event adjudication and whether the primary end point consisted of more than 1 component or included all-cause death. Estimated sample size and power were extracted from the initial protocol. We classified the justification for event rate or effect size estimation provided by the authors according to the level of evidence as follows: pilot study or phase 2 study, meta-analysis, multiple RCTs, single RCT, multiple observational studies, single observational study, unpublished data, or no source indicated.

#### Conduct and Results

If the primary outcome in the identified publication differed from the primary outcome reported initially, we documented the changes in the primary end point. These trials were not excluded from analysis. We assessed the following recruitment parameters: multicontinental location (>1 continent), number of sites, number of countries, duration of recruitment in months, and number of participants randomized. We assessed whether trials were terminated early without determining the reason for early termination. Significant refutation of the null hypothesis was prespecified and defined as a statistically significant outcome for a study per the predefined *P* value.

### Statistical Analysis

For trial description, categorical variables are presented as numbers with frequencies and continuous variables are presented as medians with IQRs. Accuracy for each trial is expressed as the ratio of the observed to estimated event rate or effect size, respectively. Linear regression was used to determine the overall accuracy of estimation across all trials and the association of this accuracy with trial design characteristics (ie, research organization type, sponsor and funding, intervention type, masking, clinical events committee, composite primary end point, primary end point included all-cause death, estimated sample size, provided justification for estimation, and power ≥90%). Data were analyzed with Prism, version 9.2.0 (GraphPad Software), or with Stata, version 17 (StataCorp). All tests were 2 tailed, and *P* ≤ .05 was considered statistically significant. No adjustment was made for multiple testing. Therefore, *P* values should not be interpreted as confirmatory but are descriptive in nature, and inferences drawn from the 95% CIs may not be reproducible.

## Results

**Figure 1.**
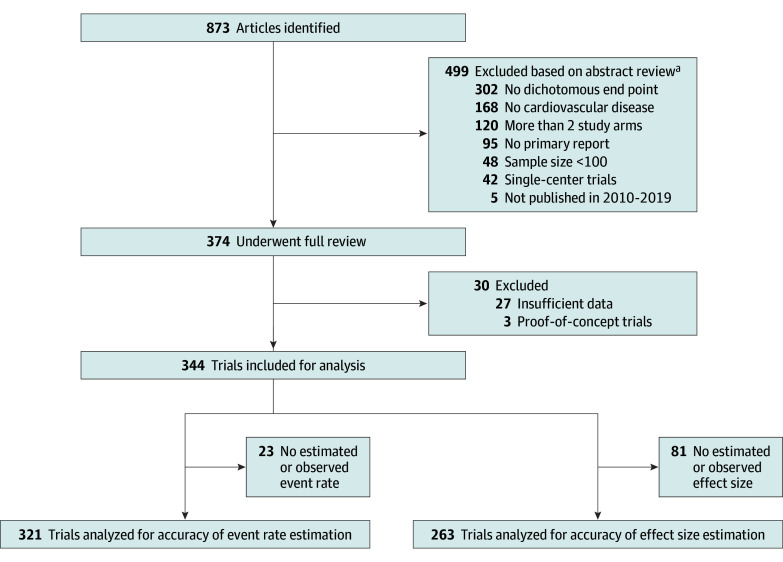
Flow Diagram of Literature Search and Screening ^a^Articles could meet more than 1 exclusion criterion.

Of the 873 publications identified, 374 underwent full review (Figure 1). After exclusion of trials with insufficiently reported data for this analysis, data from 344 trials were analyzed. Of the 344 included trials, 52 (15.1%) were published in *JAMA*, 80 (23.3%) in the *Lancet*, and 212 (61.6%) in the *New England Journal of Medicine*. The most common investigated areas were cardiovascular risk factors (149 trials [43.3%]), antithrombotic therapy (82 trials [23.8%]), heart failure (27 trials [7.8%]), or arrhythmia (24 trials [6.9%]). Trial characteristics are listed in the Table. No estimated or observed event rate was identified in 23 trials. These trials were excluded for analysis of the event rate. The majority of trials (263 [76.5%]) were designed to test for superiority of the intervention. Because noninferiority trial designs do not necessarily include an estimated effect size, the remaining 81 trials (23.5%) were not included in the analyses for effect size. Characteristics of the 321 trials for event rate analysis and the 263 superiority trials for effect size analysis are detailed in eTable 3 in Supplement 1.

**Table.** Trial Characteristics^a^

Characteristic	Values (N = 344)
Research organization type	
Academic only	186 (54.1)
Contract	158 (45.9)
Sponsor and funding	
Industry sponsored	145 (42.2)
IIT, industry co-funding	122 (35.5)
IIT, nonprofit funding	77 (22.1)
Intervention type	
Drug	186 (54.1)
Device	113 (32.8)
Other	45 (13.1)
Design	
Masking	198 (57.6)
Clinical events committee	295 (85.8)
Composite primary end point	247 (71.8)
Primary end point included all-cause death	124 (36.0)
Estimated sample size	2386 (800 to 6000)
Power ≥90%	144 (41.9)
Conduct and results	
Major change in the primary end point^b^	27 (7.8)
Multicontinental location	184 (53.5)
No. of sites	66 (22 to 245)
No. of countries	5 (1 to 20)
Early termination	42 (12.2)
Recruitment duration, mo	37 (24 to 56)
No. of randomized participants	2209 (759 to 6053)
Significant refutation of the null hypothesis	149 (43.3)

Abbreviation: IIT, investigator-initiated trial.

^a^ Unless specified otherwise, values are presented as the No. (%) of trials or the median (IQR).

^b^ Addition or removal of a primary end point from protocol to primary report.

### Trial Design, Conduct, and Results

Of the 344 trials analyzed, 186 (54.1%) were conducted by an academic research organization and 145 (42.2%) were industry sponsored. The majority (186 [54.1%]) tested a drug as the intervention. A clinical events committee for event adjudication was often included (295 trials [85.8%]) and a composite primary end point was common (247 trials [71.8%]). The median estimated sample size was 2386 (IQR, 800 to 6000). The majority of articles or protocols provided a justification for the estimation of the event rate (229 trials [71.3%]; eTable 4 in Supplement 1). Single (82 of 321 [25.5%]) or multiple (79 of 321 [24.6%]) RCTs were the most common justification provided for the estimation of event rates. For estimated effect size, more than half of trials (150 of 263 [57.0%]) did not provide a justification. The most common rationales were meta-analyses (34 trials [12.9%]), multiple RCTs (31 trials [11.8%]), and single RCTs (33 trials [12.6%]). In 18 trials (6.8%), the authors justified the effect size with the smallest clinically important difference.

The majority of trials were conducted on more than 1 continent, with a median of 66 (IQR, 22 to 245) sites. A median of 2209 (IQR, 759 to 6053) participants were recruited over a median of 37 (IQR, 24 to 56) months. The median difference in recruited participants from the estimated sample size was 6 (IQR, −1 to 67; paired *t* test: *P* = .82). Of the 302 trials that were not terminated early, the median difference in recruited participants from the estimated sample size was 8 (IQR, 0 to 72). In 27 trials (7.8%), a major change in the primary end point during trial conduct was observed.

### Event Rate Estimation

**Figure 2.**
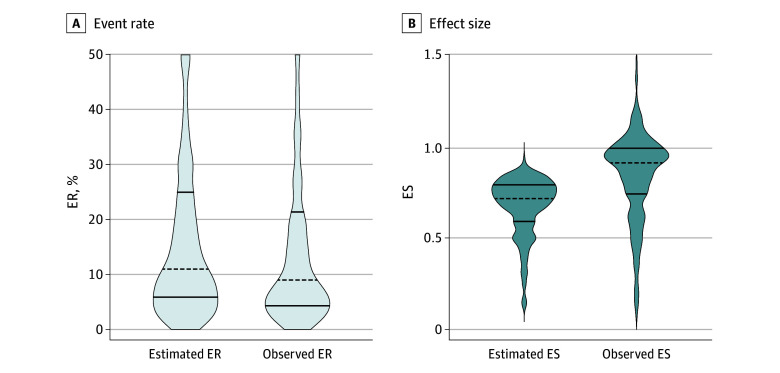
Estimated and Observed Event Rates (ERs) and Effect Sizes (ESs) Dashed lines indicate medians; solid lines indicate IQRs. A, The median estimated ER was 11.0% (IQR, 6.0%-25.0%); the median observed ER was 9.0% (IQR, 4.3%-21.4%). The mean relative deviation from the estimation was −12.3% (95% CI, −16.4% to −5.6%). B, The median estimated ES was 0.72 (IQR, 0.60-0.80); the median observed ES was 0.91 (IQR, 0.74-0.99). The mean relative overestimation of the ES was 23.1% (95% CI, 17.9%-28.3%).

**Figure 3.**
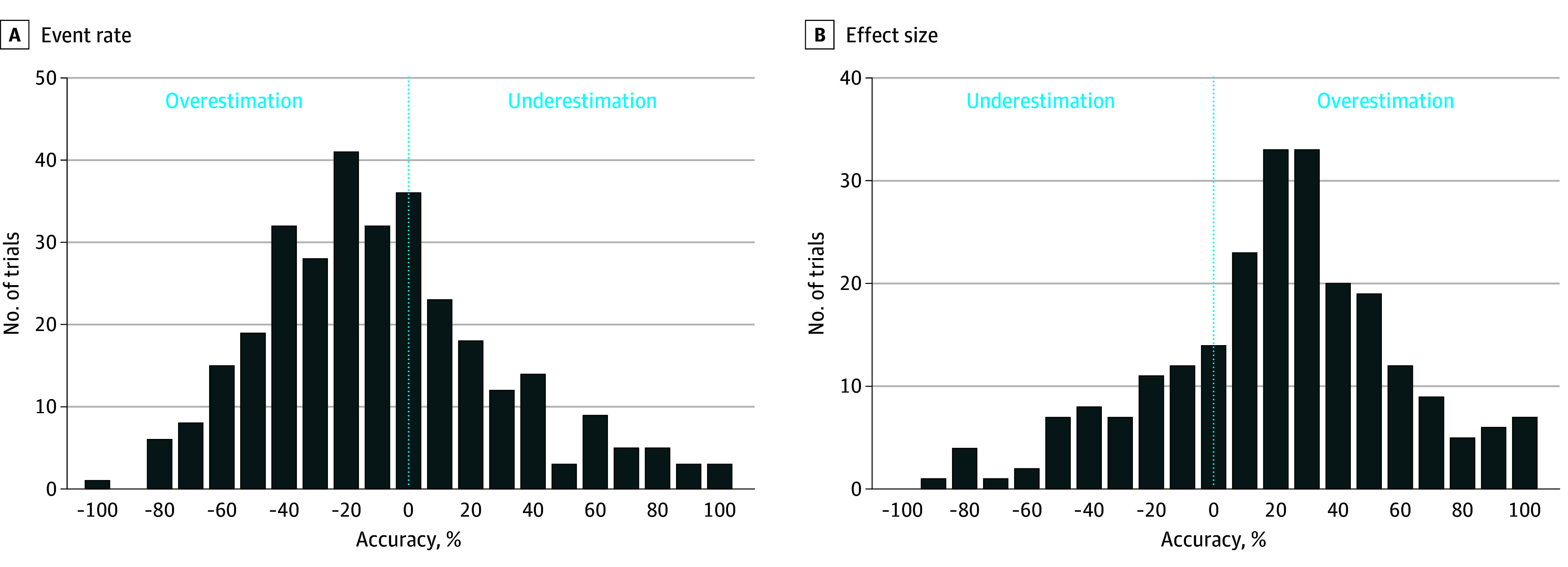
Accuracy of Event Rate (ER) and Effect Size (ES) Estimation A, Overestimation (196 trials [61.1%]) and underestimation (119 trials [37.1%]) of ER in randomized clinical trials (RCTs). Accuracy was calculated as follows: (Observed ER – Estimated ER)/Estimated ER. B, Underestimation (47 trials [17.9%]) and overestimation (216 trials [82.1%]) of ES in RCTs. Accuracy was calculated as follows: (Observed ES – Estimated ES)/Estimated ES.

**Figure 4.**
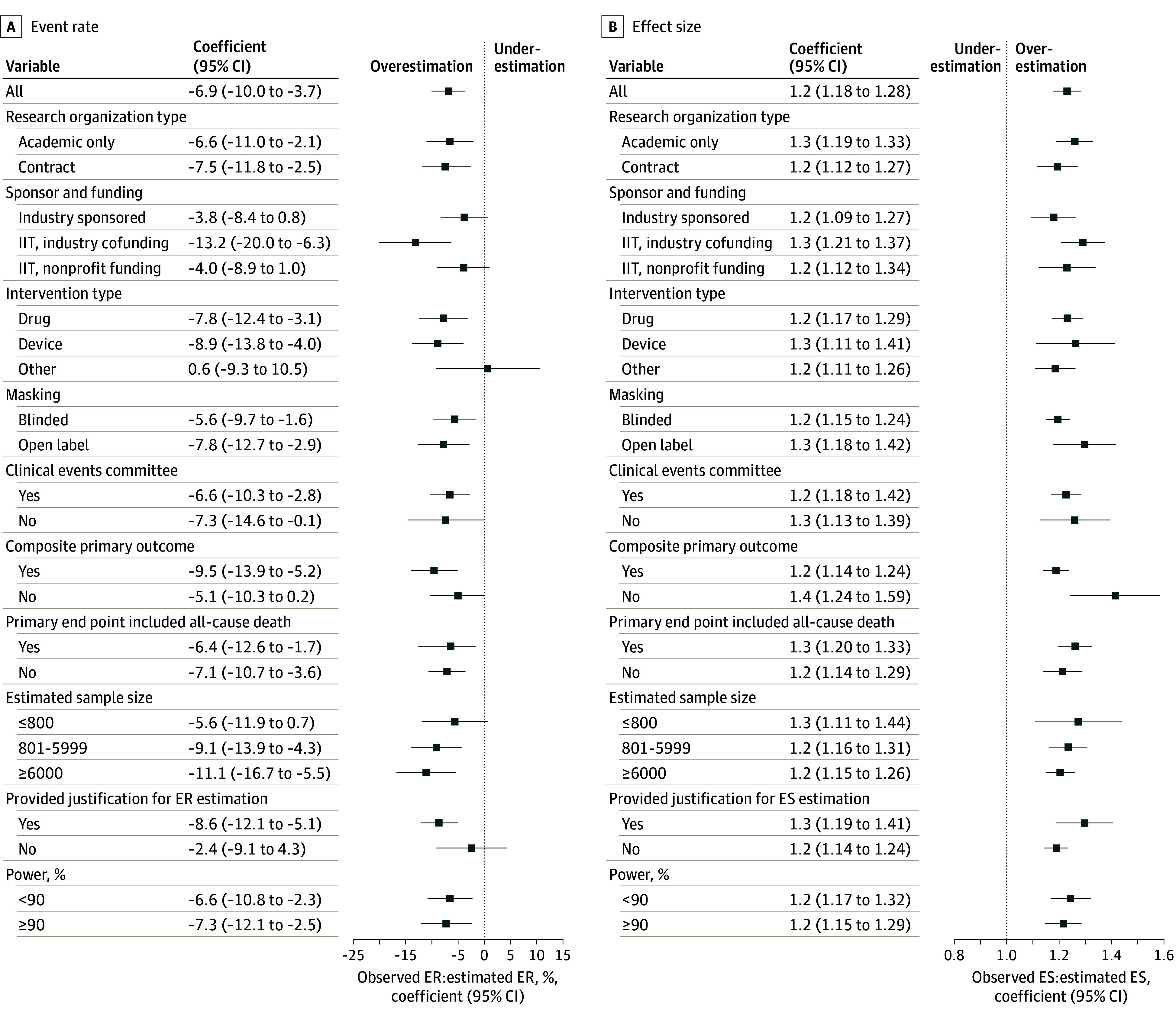
Linear Regression for Event Rate (ER) and Effect Size (ES) Estimation A, Accuracy of ER estimation. B, Accuracy of ES estimation. IIT indicates investigator-initiated trial. Error bars indicate 95% CIs.

The median observed event rate was 9.0% (IQR, 4.3% to 21.4%), which was significantly lower than the estimated event rate (11.0% [IQR, 6.0% to 25.0%]; mean relative deviation from estimation, −12.3% [95% CI, −16.4% to −5.6%]; *P* < .001). A total of 196 trials (61.1%) overestimated the event rate (Figures 2A and 3A). The overestimation was consistent across several subgroups (Figure 4A). Accuracy of event rate estimation was associated with a significant refutation of the null hypothesis (0.13 [95% CI, 0.01 to 0.25]; *P* = .03).

In a multiple linear regression model (eTable 5 in Supplement 1), device trials were independently associated with decreased accuracy of event rate estimation (14.0% reduction in accuracy [95% CI, −0.27 to −0.01]) compared with drug trials. Masking and clinical events committee installation contributed to higher event rate estimation accuracy but were not statistically significant.

### Effect Size Estimation

The median observed effect size was 0.91 (IQR, 0.74 to 0.99), which was significantly lower compared with the estimated effect size (0.72; IQR, 0.60 to 0.80; mean relative overestimation of effect size, 23.1% [95% CI, 17.9% to 28.3%]). A total of 216 trials (82.1%) overestimated the effect size. Overestimation was consistent across all subgroups (Figure 4B). In a multiple linear regression model (eTable 5 in Supplement 1), masking and use of a composite primary end point were independently associated with higher effect size estimation accuracy. Justification for effect size estimation was an independent indicator of decreased effect size estimation accuracy. Clinical events committee installation tended to contribute to higher effect size estimation accuracy but was not statistically significant. Effect size estimation accuracy was associated with significant refutation of the null hypothesis (−0.59 [95% CI, −0.82 to −0.36]; *P* < .001).

## Discussion

The main findings of this systematic review suggest that during the design of contemporary cardiovascular trials, event rates are often overestimated but event rate estimation accuracy is associated with significant refutation of the null hypothesis. We observed that 4 of 5 trials included in this review overestimated the effect size of the tested intervention. We identified several variables of trial design as independently associated with effect size estimation accuracy. The numbers of recruited participants did not significantly differ from the estimated sample sizes.

### Event Rate Overestimation

We observed overestimation of event rates in the design of contemporary cardiovascular trials included in this review. Accuracy of event rate estimation was associated with a statistically significant refutation of the primary trial hypothesis. Overestimation of event rates may lead to potential type II error and trial failure and reduce scientific impact.^7,8^ Assumptions based on data from prior studies that did not include improvement of medical treatment and prognosis over time and a decrease in event rates might partially explain this overestimation. Optimistic assumptions may seem to facilitate trial feasibility, such as through smaller sample size. Future trial designs may include assumptions on improvement on medical treatment to anticipate a decrease in event rates and avoid underpowered trial designs.

The overestimation observed was consistent across several subgroups of trials. Compared with drug trials, event rate estimation accuracy was decreased in device trials. This finding is consistent with 2 smaller studies of randomized clinical device trials that demonstrated a higher relative difference of the difference in event rate estimation we observed.^9,10^ This study suggests that those findings, which are centered on specific fields of cardiovascular trials, are systematic and overestimation is common in cardiovascular RCTs.

No association of event rate estimation accuracy with a provided justification was observed. However, justification of event rate estimation was diverse. Approximately 1 in 4 studies relied on observational data. Clinical trials often have been criticized as being noninclusive.^11^ Highly selective trial populations might partially contribute to overestimation of event rates because, for example, observational data may include older patients or patients with advanced kidney disease who may have higher event rates but are often underrepresented in clinical trials.

More than 1 in 4 trials in this review did not provide justification for event rate estimation. Because overestimation of event rates may lead to underpowered trial design, researchers should be encouraged to justify the event rate estimation. In this review, we observed that clinical events committee installation tended to increase event rate estimation accuracy. Clinical events committees use standardized methods for event adjudication and reliably identify events in cardiovascular trials, often identifying more events than site investigator report.^12^

### Effect Size Overestimation

This study identified a substantial overestimation of effect sizes in contemporary RCTs in cardiovascular medicine: 4 of 5 trials overestimated the effect size of the tested intervention. This optimism bias is consistent with observations in other subspecialties of medicine that demonstrated a significant overestimation of effect size,^13,14^ which might be even more pronounced in oncology studies.^15,16^

An average overestimation of effect size in clinical research is often found and can be justified by the principle of clinical equilibrium—that is, a high degree of uncertainty as to whether the intervention will be superior prior to trial conduct. If there is an excessively high degree of certainty about the intervention, ethical concerns on study conduct would arise and challenge participant recruitment.^17,18^ Djulbegovic et al^19,20,21^ observed a symmetrically distributed effect in favor of the intervention in RCTs with slight superiority of the intervention. In superiority trials, researchers aim to demonstrate an effect size in favor of the intervention for a new study. Thus, an overall overestimation of effect size seems inevitable.

Masking reduces bias in clinical trials.^22^ In this review, masking was associated with higher effect size estimation accuracy. Masking may lead to more valid estimation of the effect of an intervention in a trial when conducted. However, treatment effect size estimates are often larger in trials that lack masking.^23^ Use of a composite primary end point was associated with higher accuracy, which may be explained by a decreased variance of an increased sample size. However, a previous study suggested that primary end points with multiple components facilitate the misinterpretation of trial results^24^: Components of the primary end point may have clinically meaningful differences and should be interpreted with caution. More than half of the trials in this study did not provide a justification for effect size estimation; however, this absence was independently associated with higher effect size estimation accuracy. It does not seem plausible that a lack of data would increase effect size estimation accuracy. Lack of adequate control for multiple comparisons may be responsible for this association. We observed that 1 of 8 trials in this review justified the estimation with observational data. Associations from observational studies may imply causation but do not prove causation, which might in part explain the overestimation. Thus, this factor should be considered an indicator (rather than a causal factor) of high effect size estimation accuracy.

### Perspective

Trial investigators estimate event rates and effect sizes from a sincere evaluation grounded in both data and their expertise. Reasons for the overestimation of event rates and effect sizes observed here were not sufficiently identified. Possible factors contributing to this observation include the absence of robust data to inform event rate estimation, more restrictive criteria for trial inclusion resulting in a lower-risk population and subsequently lower event rates, advancements in clinical care lowering event rates over time, preclinical research that may overestimate the effect size, and financial constraints or feasibility concerns prompting the restriction of sample size through optimal assumption of event rates and effect sizes. In this study, accuracy of event rate and effect size estimation was associated with significant refutation of the null hypothesis.

### Limitations

This study has some limitations. First, the restriction to studies published in 1 of 3 specific journals limits the generalizability of the results: (1) these insights might be different in other disciplines of medicine and (2) trials with a statistically significant result are published more frequently in these journals.^7^ Thus, overestimation of event rates and effect sizes in trials published in less cited journals may even be higher. However, many positive trials are published in highly cited specialty journals. A focus on event types specific for diseases may be helpful in identifying areas to correct event rate and effect size estimation inaccuracy. However, limited subgroup size restricts the ability to detect smaller associations. Second, this study only included trials that indicated estimated and observed event rates or effect sizes in their published data. This criterion may have introduced a selection bias: adherence to standardized procedures including reporting guidelines may be associated with both estimation accuracy and trial success. Third, changes in the primary end point during trial conduct may have led to less accurate results when estimated and observed variables were compared. Fourth, because we analyzed data from trials with dichotomous outcomes, these findings cannot be extrapolated to trials with continuous outcomes. The dichotomization of variables in clinical research has been criticized^25^ and often does not reflect the biological continuum or the relevance of an outcome to the patient.

## Conclusions

In this systematic review of contemporary cardiovascular RCTs, the event rates of the primary end point and effect sizes of an intervention were frequently overestimated. This overestimation may have contributed to the inability to adequately test the trial hypothesis.

## Notes

Supplement 1. **eTable 1.** Results of the Literature Search **eTable 2.** Detailed Inclusion and Exclusion Criteria **eTable 3.** Characteristics for the Trials Included for the Analyses of Event Rates or Effect Size Ratios **eTable 4.** Justification of Estimation **eTable 5.** Multiple Linear Regression: Factors Associated With Accuracy of Estimation of Event Rates or Effect Sizes **eAppendix.** Conversion Formulas in Harmonization Process
eReference

Supplement 2.
Data Sharing Statement
