# Supplementary material for: Accuracy of Event Rate and Effect Size Estimation in Major Cardiovascular Trials: A Systematic Review
Source: JAMA Netw Open. 2024 Apr 30;7(4):e248818. doi: 10.1001/jamanetworkopen.2024.8818 (PMC11061773; doi:10.1001/jamanetworkopen.2024.8818)
Supplement: Supplement 1. — eTable 1. Results of the Literature Search eTable 2. Detailed Inclusion and Exclusion Criteria eTable 3. Characteristics for the Trials Included for the Analyses of Event Rates or Effect Size Ratios eTable 4. Justification of Estimation eTable 5. Multiple Linear Regression: Factors Associated With Accuracy of Estimation of Event Rates or Effect Sizes eAppendix. Conversion Formulas in Harmonization Process eReference [file jamanetwopen-e248818-s001.pdf]

Supplementary Online Content

Olivier CB, Struß L, Sünnen N, et al. Accuracy of event rate and effect size estimation in major cardiovascular trials: a systematic review. *JAMA Netw Open*. 2024;7(4):e248818. doi:10.1001/jamanetworkopen.2024.8818

eTable 1. Results of the Literature Search
eTable 2. Inclusion and Exclusion Criteria
eTable 3. Characteristics for the Trials Included for the Analyses of Event Rates or Effect Sizes
eTable 4. Justification of Estimation
eTable 5. Multiple Linear Regression: Factors Associated With Accuracy of Estimation of Event Rates or Effect Sizes
eAppendix. Conversion Formulas in Harmonization Process
eReference

This supplementary material has been provided by the authors to give readers additional information about their work.

© 2024 Olivier CB et al. *JAMA Network Open.*

eTable 1. Results of the Literature Search

#	Searches	Results	Annotations
	"new england journal of medicine".jn.	79640	Selected journals
2	jama.jn.	72624	
3	lancet.jn.	136984	
4	1 or 2 or 3	289248	
5	limit 4 to yr="2010 - 2019"	41327	Selected journals from 2010-2019 at date of search
6	exp randomized controlled trial/	515712	Publication type RCT incl. narrower terms equivalence trial (from 2018), pragmatic clinical trial (from 2014)
7	5 and 6	3054	Selected journals from 2010-2019 at date of search AND RCT
8	exp Cardiovascular Diseases/	2401641	MeSH CVD (MeSH incl. narrower terms from the hierarchy)
9	7 and 8	873	Selected journals from 2010-2019 at date of search AND RCT AND CVD MeSH terms
10	remove duplicates from 9	873	

© 2024 Olivier CB et al. *JAMA Network Open.*

eTable 2. Inclusion and Exclusion Criteria

**Inclusion criteria**	• Randomized controlled trial<br>• Multicenter trial<br>• Cardiovascular disease<br>• Published between 01.01.2010 and 31.12.2019<br>• Cardiovascular endpoint<br>• Sample size >100<br>• Estimated event rate or effect size provided
**Exclusion criteria**	• Surgical intervention only<br>• Study arms >2<br>• Exploratory study

© 2024 Olivier CB et al. *JAMA Network Open.*

eTable 3. Characteristics for the Trials Included for the Analyses of Event Rates or Effect Sizes

Characteristic	Event Rate		Effect Sizes	
	No. 321	(%) (100)	No. 263	(%) (100)
**General**				
**Type of research organization**				
**Academic only**	177	(55.1)	155	(58.9)
**Contract**	144	(44.9)	108	(41.1)
**Sponsor and funding**				
**Industry-sponsored**	133	(41.4)	98	(37.3)
**IIT, industry co-funding**	113	(35.2)	96	(36.5)
**IIT, non-profit funding**	75	(23.4)	69	(26.2)
**Type of intervention**				
**Drug**	176	(54.8)	153	(58.2)
**Device**	108	(33.6)	71	(27.0)
**Other**	37	(11.5)	39	(14.8)
**Design**				
**Masking**	182	(56.7)	160	(60.8)
**Clinical events committee**	279	(86.9)	220	(83.7)
**Composite primary outcome**	235	(73.2)	211	(80.2)
**Primary endpoint included all-cause death**	117	(36.4)	98	(37.3)
**Estimated sample size, median (IQR), n**	2406	(885;6000)	2500	(706;6850)
**Provided justification for estimation**	229	(71.3)	113	(43.0)
**Power ≥90%**	134	(41.7)	112	(42.6)
**Conduct and results**				
**Major change of the primary endpoint***	25	(7.8)	23	(8.7)
**Multi continental location**	150	(46.7)	184	(53.5)
**Number of sites, median (IQR), n**	65.5	(22;245)	74	(24;245)
**Number of countries, median (IQR), n**	4.5	(1;20)	5	(1;20)
**Early termination**	40	(12.5)	37	(14.1)
**Duration of recruitment, median (IQR), months**	36.5	(24;56)	39.0	(27.0;57.3)
**Randomized participants, median (IQR), n**	1082	(397;2741)	2278	(691;6983)
**Significant refutation of the null hypothesis**	141	(43.9)	85	(32.3)

*Addition or removal of a primary endpoint from protocol to primary report; Abbreviations: IIT, investigator-initiated trial, IQR, interquartile range.

© 2024 Olivier CB et al. *JAMA Network Open.*

eTable 4. Justification of Estimation

	Event Rate		Effect Sizes	
**Justification for estimation (Multiple answers possible)**	**No. 321**	**(%) (100)**	**No. 263**	**(%) (100)**
**Minimal clinically important difference**	Not applicable		18	(6.8)
**Pilot study or Phase 2 study**	9	(2.8)	5	(1.9)
**Meta-analysis**	42	(13.1)	34	(12.9)
**Multiple RCT**	79	(24.6)	31	(11.8)
**Single RCT**	82	(25.5)	33	(12.5)
**Multiple observational studies**	49	(15.3)	16	(6.1)
**Single observational study**	40	(12.5)	16	(6.1)
**Unpublished data**	13	(4.0)	5	(1.9)
**Not provided**	92	(28.7)	150	(57.0)

© 2024 Olivier CB et al. *JAMA Network Open.*

eTable 5. Multiple Linear Regression: Factors Associated With Accuracy of Estimation of Event Rates or Effect Sizes

	Event rate *		Effect sizes *	
**Characteristic**	Coefficient (95% CI)	p-value	Coefficient (95%-CI)	p-value
**General**				
Contract research organization	-0.018 (-0.178 to 0.142)	0.824	-0.032 (-0.398 to 0.333)	0.863
Sponsor and funding				
Industry-sponsored	reference		reference	
IIT, industry co-funding	-0.025 (-0.179 to 0.142)	0.761	0.106 (-0.329 to 0.541)	0.631
IIT, non-profit funding	-0.012 (-0.188 to 0.213)	0.906	-0.015 (-0369 to 0.338)	0.932
Type of intervention				
Drug	reference		reference	
Device	-0.140 (-0.268 to -0.011)	0.033	-0.166 (-0.468 to 0.136)	0.279
Other	-0.065 (-0.258 to 0.127)	0.507	-0.323 (-0.686 to 0.040)	0.081
**Design**				
Masking	0.080 (-0.043 to 0.203)	0.199	-0.482 (-0.763 to 0.200)	0.001
Clinical events committee	0.130 (-0.038 to 0.298)	0.130	0.270 (-0.051 to 0.590)	0.098
Composite primary endpoint	-0.044 (-0.173 to 0.084)	0.498	-0.297 (-0.588 to -0.006)	0.045
Primary endpoint included all-cause death	0.062 (-0.046 to 0.170)	0.259	-0.174 (-0.422 to 0.073)	0.166
Estimated sample size				
≤800	reference		reference	
801-5999	-0.093 (-0.232 to 0.046)	0.190	-0.039 (-0.325 to 0.247)	0.790
≥6000	-0.026 (-0.199 to 0.148)	0.771	-0.060 (-0.408 to 0.288)	0.736
Provided justification for estimation	-0.035 (-0.154 to 0.084)	0.567	0.315 (0.078 to 0.552)	0.009
Power: ≥90%	0.032 (-0.075 to 0.139)	0.555	-0.039 (-0.286 to 0.208)	.0754

Abbreviations: IIT, investigator-initiated trial, IQR, interquartile range.
* Observed/Estimated

© 2024 Olivier CB et al. *JAMA Network Open.*

eAppendix. Conversion Formulas in Harmonization Process

A)

$$HR = \frac{ln\,(\frac{(100 - ERi)}{100})}{ln\,(\frac{(100 - ERc)}{100})}$$

HR= Hazard Ratio; ERi= Event Rate intervention; ERc= Event Rate control

B)

$$OR = \frac{(\frac{ERi}{100 - ERi})}{(\frac{ERc}{100 - ERc})}$$

OR= Odds Ratio; ERi= Event Rate intervention; ERc= Event Rate control

eReference

Angermann CE, Gelbrich G, Stork S, et al. Effect of Escitalopram on All-Cause Mortality and Hospitalization in Patients With Heart Failure and Depression: The MOOD-HF Randomized Clinical Trial. JAMA. 2016;315(24):2683-2693. doi:10.1001/jama.2016.7635

© 2024 Olivier CB et al. *JAMA Network Open.*
