# Supplementary material for: Accuracy of Event Rate and Effect Size Estimation in Major Cardiovascular Trials: A Systematic Review
Source: JAMA Netw Open. 2024 Apr 30;7(4):e248818. doi: 10.1001/jamanetworkopen.2024.8818 (PMC11061773; doi:10.1001/jamanetworkopen.2024.8818)
Supplement: Supplement 2. — Data Sharing Statement [file jamanetwopen-e248818-s002.pdf]

# Data Sharing Statement

Olivier. Accuracy of Event Rate and Effect Size Estimation in Major Cardiovascular Trials. *JAMA Netw Open*. Published April 30, 2024. doi:10.1001/jamanetworkopen.2024.8818

**Data**
**Data available:** No. The data that support the findings of this study are available from the corresponding author (CBO) upon reasonable request. Neither this review, nor this review´s protocol, nor any amendments were registered.
